# Expression of Concern: Association of FcεRIβ polymorphisms with risk of asthma and allergic rhinitis: Evidence based on 29 case-control studies

**DOI:** 10.1042/BSR-20180177_EOC

**Published:** 2020-07-22

**Keywords:** allergic diseases, FcεRIβ, genetic model, Meta-analysis, polymorphism

The Editorial Office has been made aware of potential issues surrounding the scientific validity of this paper, as commented on in the correspondence “Comments on ‘Association of FcεRIβ polymorphisms with risk of asthma and allergic rhinitis: evidence based on 29 case-control studies’”, and therefore has issued an expression of concern to notify readers to potential errors in the conclusions. The authors were invited to respond to the comments made, but have not responded.

